# A feasibility study of psychological first aid as a supportive intervention among police officers exposed to traumatic events

**DOI:** 10.3389/fpsyg.2023.1149597

**Published:** 2023-03-13

**Authors:** Steve Geoffrion, Marie-Pierre Leduc, Elody Bourgouin, François Bellemare, Valérie Arenzon, Christine Genest

**Affiliations:** ^1^Trauma Studies Center, Research Centre of the University Institute in Mental Health of Montreal, Montreal, QC, Canada; ^2^School of Psychoeducation, Université de Montréal, Montreal, QC, Canada; ^3^Department of Psychology, Université de Montréal, Montreal, QC, Canada; ^4^Department of Nursing, Université de Montréal, Montreal, QC, Canada

**Keywords:** psychological first aid, police work, feasibility, applied psychology, demand, acceptabilitity, practicability

## Abstract

**Introduction:**

Police officers are often exposed to traumatic events, which can induce psychological distress and increase the risk of developing post-traumatic stress injuries. To date, little is known about support and prevention of traumatic events in police organizations. Psychological first aid (PFA) has been promoted as a promising solution to prevent psychological distress following exposure to a traumatic event. However, PFA has not yet been adapted to policing reality, let alone to the frequent exposure to traumatic events faced by this population. This study aimed to evaluate the feasibility of PFA as an early intervention for the prevention of post-traumatic stress injuries among police officers in Quebec, Canada. Specifically, the objectives were to evaluate: (1) the demand. (2) the practicality, and (3) the acceptability of PFA in a police organization.

**Methods:**

A feasibility study was conducted to evaluate the implementation of PFA among Quebec’s provincial police force. To do so, 36 police officers participated in semi-structured interviews between October 26th, 2021, and July 23rd, 2022. Participants were comprised of responders (*n* = 26), beneficiaries (*n* = 4) and managers (*n* = 6). Interviews were transcribed, coded, and evaluated according to a thematic analysis.

**Results:**

Eleven themes emerged from participants’ responses. Results suggested that PFA met individual and organizational needs. References were also made regarding the impacts of this intervention. Moreover, participants provided feedback for improving the implementation and sustainability of a PFA program. All three groups of participants shared similar thematic content.

**Discussion:**

Findings revealed that implementation of a PFA program in a law enforcement agency was feasible and could be accomplished without major issues. Importantly, PFA had beneficial consequences within the organization. Specifically, PFA destigmatized mental health issues and renewed a sense of hope among police personnel. These findings are in line with previous research.

## 1. Introduction

Throughout their careers, police officers are often exposed to traumatic events (TEs) such as accidents, physical assaults, or suicides. For instance, over 90% of Canadian public safety personnel (PSP; police, firefighters, paramedics, dispatchers, and correctional workers) have been involved in a serious transportation accident, and over 90% have observed abrupt or violent deaths (Carleton et al., 2019). Traumatic events can cause psychological distress among police officers, hence increasing the risk of developing post-traumatic stress injuries (PTSI; Public Safety Canada, 2018;^1^ Carleton et al., 2019; Anderson et al., 2020). PTSI is a non-clinical term used to assess the consequences associated with traumatic events. It refers to a variety of mental health disorders, including post-traumatic stress disorder (PTSD), depression, anxiety, as well as other related issues such as physiological arousal, substance abuse, alcoholism, social isolation, absenteeism, domestic violence, suicidal ideations, and attempts (Martin et al., 2009; Ballenger et al., 2011; Stanley et al., 2016; Carleton et al., 2019; Anderson et al., 2020; Public Safety Canada, 2022^2^). Research has shown that police officers are at high risk for developing PTSI. For instance, up to 19.6% of provincial police officers and up to 30% of Royal Canadian Mounted Police met the diagnostic criteria for post-traumatic stress disorder (Carleton et al., 2018a,b), while the lifetime prevalence in the Canadian general population is 9.2% (Van Ameringen et al., 2008). Additionally, research has demonstrated that police officers have higher rates of diagnosed mental disorders (Carleton et al., 2018a,b) and suicide ideation than the general population (Stanley et al., 2016; Carleton et al., 2018a,b).

To date, empirical evidence about prevention practices and consequences of TEs in police settings remains limited. Existing research on PTSI mainly focuses on treatments and their efficacy (Corthesy-Blondin et al., 2021). In practice, evidence-based treatment resources for PTSI are scarce and are mostly located in large urban centers. Hence, police officers in remote areas have limited access to these services (Public Safety Canada, 2022). It is also important to consider that mental health disorders are largely stigmatized within police culture. Thus, trauma exposed officers may avoid seeking external resources (Soomro and Yanos, 2019; Wheeler et al., 2021; Public Safety Canada, 2022). A recent study by Hofer and Savell (2021) revealed that police officers were reluctant to seek mental health care due to fear of being perceived as weak, untrustworthy, or unreliable. Additional research has demonstrated that stigma increases the likelihood of PTSI related reactions among police officers (Geoffrion et al., 2017; Soomro and Yanos, 2019).

Currently, experts in post-traumatic intervention recommend the administration of psychological first aid (PFA) to prevent psychological distress after a TE (World Health Organization [WHO], 2011;^3^ Fox et al., 2012; Shultz and Forbes, 2014). PFA was initially created for humanitarian crisis intervention in the aftermath of a natural disaster or terrorist attack. Yet, the last decade has seen a widespread use of PFA across different contexts (Shultz and Forbes, 2014; Hermosilla et al., 2022). PFA is an evidence-informed, modular approach designed to promote safety, calmness, self-efficacy, connectedness, and hope among survivors of TEs (Hobfoll et al., 2007; Kouvatsou et al., 2022). Additionally, PFA aims to reduce initial distress, enhance the ability to manage stress reactions, minimize psychopathological risk factors, and promote short and medium-term adaptive functioning (Brymer et al., 2006, p. 5). It is comprised of empirically validated interventions known to effectively reduce anxiety, depression, and post-traumatic stress symptoms (Hermosilla et al., 2022). Unlike psychotherapy, the administration of PFA does not require a mental health professional, but rather a trained PFA responder. Specifically, aid is characterized by empathetic support and coaching of various coping strategies (Brymer et al., 2006, p. 5; Inter-Agency Standing Committee [IASC] 2007). Accordingly, PFA’s core actions include: (1) the *contact and engagement*, which refer to initiating or responding to recipients contact in a non-intrusive and caring way, (2) the enhancement of recipients *safety and comfort*, (3) the *stabilization* of the recipients, as they can be emotionally overwhelmed, (4) *the information gathering* of recipients needs and concerns in order to tailor the PFA intervention, (5) the *practical assistance*, which refers to the applied response to these needs and problems, (6) the connection of recipients with social support (e.g., family members, friends, and community resources), (7) the presentation of *information* on stress and *coping* strategies to reduce distress, and (8) the linkage of recipients with collaborative services (e.g., employee aid program) for their immediate and future needs. Importantly, providers are required to remain flexible to ensure that recipients’ needs and concerns are appropriately addressed (Brymer et al., 2006, p. 19).

There is a need for additional research on the suitability of PFA for law enforcement agencies. Although police officers are frequently exposed to TEs, PFA interventions have not yet been adapted for their reality. Evidence provided by a recent systematic review suggests that PFA interventions improve mental health and psychosocial well-being among trauma exposed individuals (Hermosilla et al., 2022). Nonetheless, the quality of these findings remain poor due to methodological variability (e.g., inconsistent intervention components, insufficient evaluation methodologies) and bias among studies. Altogether, research has yet to determine how to implement a suitable and effective PFA program within police organizations.

The current study aimed to evaluate the feasibility of PFA as an early intervention designed to prevent the development of PTSI following exposure to TEs among police officers in Quebec, Canada. Specifically, the study aimed to evaluate: (1) the demand, (2) the practicality, and (3) the acceptability of PFA in a police organization.

## 2. Materials and methods

### 2.1. Study design

A feasibility study (Bowen et al., 2009) was conducted to determine if PFA interventions could an acceptable post-traumatic intervention among police officers. To develop a comprehensive intervention adapted for policing, a collaboration was established between researchers and police organization. The partnership aimed to evaluate whether a peer delivered PFA program meets the needs of officers at risk of developing PTSI. The project has received ethics approval from the CIUSSS de l’Est-de-l’Île-de-Montréal ethics committee (#2022–2,673). Before starting the study, informed consent was obtained from all participants. The consent form explained the overall procedure of the study and its intentions. To ensure confidentiality, a unique identifying number was attributed to each signed consent form. No monetary compensation was provided.

### 2.2. Context

The Sûreté du Québec (SQ) is comprised of 5,697 provincial police officers and is considered the largest police force in the province of Quebec. Overall, it serves 1,039 provincial municipalities. Along with the prevention and suppression of crime, its principal goals are the maintenance of peace, order, and public safety. From an institutional standpoint, it is the SQ’s duty to support the police force, plan significant police operations, protect the credibility of state institutions, and guarantee the safety of the transportation networks under Quebec’s jurisdiction. Additionally, it gives other police forces access to a shared intelligence network to fight against crime (Sûreté du Québec, n.d.).^4^ It is typically understood that policing places officers at high risk of repeated exposure to traumatic events. Considering the existent policing culture and the stigma within the organization, few officers are inclined to seek help when needed. In 2017, five officers from the SQ committed suicide (Lachance, 2018). In response to this series of unfortunate events, the organization sought to implement new resources for its employees. Accordingly, PFA was considered as a potentially adequate intervention program for police officers.

The present study is part of a larger initiative by the Canadian government to reduce PTSI among public safety workers (Public Safety Canada, 2022). It was carried out in two phases. For each phase, decisions were made in collaboration with the SQ. The Psychological Health Prevention Team and SQ’s Human Resources were responsible for the deployment and monitoring of the PFA program within the organization.

**Phase One: Preparation**. The preparation phase of the project focused on adapting the PFA intervention to policing context. The first action was to target the SQ units for this project. Guided by the research team, the SQ selected units whose workers were frequently exposed to TE. Then, the principal investigator (PI) worked with key members of the SQ’s training department to tailor the PFA training to the specific characteristics of the community and its members. Collaborative work was also undertaken to develop and adjust data collection tools (e.g., interview grids). Additionally, the selection criteria were established based on the recommendations of the PFA Implementation Guidelines for Workplaces at Risk (Forbes et al., 2011). Once the recruitment of responders was completed, the PI delivered the PFA training.

**PFA Training**. Participants in the PFA responders group received an adapted version of the National Child Traumatic Stress Network (NCTSN) PFA program (Brymer et al., 2006). The training was comprised of the eight PFA Core Actions and different psychoeducational concepts about trauma and support. Moreover, it included clinical vignettes and role-playing exercises, as well as short video clips about post-traumatic reactions, PFA administration, and victims’ typical needs in the hours after a traumatic event. Considering that these participants were not previously trained in mental health, the PFA training included a detailed section on PTSD, anxiety, and depression reactions, as well as the theoretical model of the development and maintenance of trauma proposed by Foa et al. (1989) and Mowrer (1960). Furthermore, as a component of practical assistance (PFA’s 5th action), the instructor taught the NCTSN’s problem-solving techniques for psychological recovery (Berkowitz et al., 2010). After the training, responders were given several tools (i.e., documentation) to facilitate the integration of PFA notions and their administration. This toolbox included the PFA administration guide, a checklist, action tracking grids and a worksheet to practice the problem-solving technique. It also provided documentation intended for beneficiaries (i.e., information on reactions following TEs and strategies to cope with them), as well as a list of help resources.

Due to the COVID-19 pandemic and the fact that the participants were spread across the province of Quebec, the training took place virtually (e.g., ZOOM) and synchronously. It consisted of 2 blocks of 3.5 h of training to cover the entire approach as well as a 2-h refresher course held 6 months after the initial training. Throughout the project, participants trained as responders were supervised through monthly co-development sessions where they could discuss PFA intervention, address preoccupations, and offer suggestions for the implementation of the program.

**Phase Two: Implementation and feasibility assessment**. The second phase of the study began with the implementation of the PFA program in the targeted units (i.e., offer information on the program and promote its use through emails and promotional posters in police stations). Then, the feasibility assessment of the program was conducted.

**PFA Intervention**. The PFA intervention proposed by the National Child Traumatic Stress Network (NCTSN; Brymer et al., 2006) has been adapted to the reality of police officers. It is important to mention that the adaptation of PFA was based on authors’ experience with police officers seeking psychotherapy services at the Montreal Trauma Studies Center. Moreover, a similar project was conducted with paramedics (see Tessier et al., 2021). First, the context in which PFA could be used was broadened. In addition to post-disaster scenes, PFA could also be offered in response to isolated TEs, repeated exposures to TEs and, for any job-related distress (e.g., depressive mood, anxiety, stress, exhaustion, insomnia, etc.), excluding labor relations issues (e.g., tension with a colleague or superior). Second, we extended the period during which PFA could be delivered. Rather than being restricted to the immediate aftermath of a TE, PFA could be offered at any time. In fact, PFA became the first intervention to be offered after a TE or upon request for support. Third, we have modified who could deliver the PFA. Rather than being offered by mental health professionals or disaster response workers, they were offered by PFA trained police officers. Fourth, the field operations guide, and the training were translated to French. Fourth, the clinical vignettes used for training and practice were adapted to a policing context. Specifically, the scenario described a police officer intervening in a context of domestic violence instead of a context of a natural disaster. Lastly, a PTSD screening could be conducted using the five-item questionnaire developed by Prins et al. (2016). The adapted guide and training for police settings are available upon request to the first author.

### 2.3. Sampling strategy and participants.

Three profiles of participants have been sought: PFA responders (or peer helpers), PFA beneficiaries and informed managers. The participants were recruited using the snowball sampling technique, thus producing a convenience sample. To be part of the study, certain inclusion criteria had to be met. First, PFA responders had to be first level managers (e.g., supervisors for new recruits or investigation team leaders). Since first level managers are mandated to provide psychological support to their colleagues and promote psychological health after a highly stressful event (e.g., authorize a break or a return home), the SQ chose these managers to be trained as PFA responders. Second, as recommended by the PFA field operation guide (Brymer et al., 2016), responders had to possess good interpersonal skills, good communication skills, and had to be psychologically fit. The organization’s human resources department conducted interviews to determine whether participants met these criteria. PFA beneficiaries had to be members of the police force (excluding civilian employees) that received PFA from a trained responder during the study. Managers had to possess basic knowledge about the PFA program and be assigned to units where responders worked. Since the PFA feasibility study was not implemented across the entire organization, the aforementioned inclusion criteria were selected to ensure that interview participants were indeed involved in the PFA program. Recruitment started on 08-17-2021 and ended on 06-21-2022. Empirical saturation was reached with a final sample of 26 PFA responders, 4 beneficiaries and 6 managers; 5 participants were female, and 31 were male.

Initially, 37 PFA responders were selected and received the training. During the study, 8 of them ceased to act as responders reporting not feeling psychologically fit to offer their support or claiming not having enough time at their disposal. Of the 28 active PFA responders, 26 accepted the interview. As for PFA beneficiaries, out of 31 possible participants, only 4 of them accepted the interview. This low recruitment rate could be partly associated with issues relating to confidentiality and, by extension, stigma surrounding the use of psychological assistance in the police environment. Some responders reported, especially at the beginning of the project, that they had offered PFA informally to avoid stigmatization. Thus, some beneficiaries did not realize that they had received PFA and therefore did not feel targeted by the general call inviting them to participate in the interview. Moreover, PFA responders reported not having complied with the demand to invite beneficiaries to participate in the study (e.g., snowball sampling) because of confidentiality concerns for beneficiaries’ identities. It is also plausible that some beneficiaries did not feel comfortable talking about personal matters to someone outside their workforce or that they were worried about being labeled as having mental problems or having needed psychological help. As for managers, they had to be assigned to one of the units where responders worked and they had to possess basic knowledge about the PFA program. However, out of 9 possible participants, only 3 satisfied the last criterion (i.e., they did not know what the training or the intervention consisted of, they were not aware of any activity or impact related to the PFA program).

### 2.4. Data collection

Data collection included semi-structured interviews conducted with all the three groups of participants. Prior to the start of each interview, the research coordinator explained the study and secured an informed written consent from all participants. The interviews were conducted between 10-26-2021 and 06-23-2022. Responders had 6 to 12 months to deliver interventions before being interviewed by the research team. Because participants were dispersed across the province, interviews were conducted by phone. Participants were asked to be in a private and peaceful location. Participants were contacted while on-duty and/or off duty. Current evidence indicates that telephone interviews do not result in inferior data quality compared to in-person interviews (Novick, 2008). On average, interviews lasted 25 min with PFA responders, 35 min with beneficiaries, and 23 min with managers. The average for the three groups was 28 min. All interviews were audio recorded and then transcribed for analysis purposes. No sociodemographic data was collected from participants to ensure their anonymity.

### 2.5. Measures

**Interview**. Semi-structured interview grids were co-created with the SQ to get participants’ perspectives on the demand (objective 1), practicability (objective 2), and accessibility (objective 3) of the PFA program. Based on authors’ previous involvement in studies with public safety personnel (Tessier et al., 2021), the research team developed a first draft of the interview grid. The initial draft was submitted to collaborators within the SQ who are responsible for the implementation of the PFA program. During a subsequent meeting, collaborators from the SQ provided their feedback. Their suggestions were integrated into the finalized interview grid that was used for the present study. Before interviewing participants, the interview grid was pre-tested by human resources actors involved in the PFA program. No modifications to the interview were necessary. As such, all three groups of participants were asked to comment on the PFA’s relevance, appropriateness, satisfaction, and suitability/fit. Participants were also asked to provide feedback regarding barriers and facilitators to the program’s implementation. Finally, PFA responders were asked to comment on their experience as peer helpers (e.g., satisfaction, concerns, impacts, etc.), while beneficiaries were asked to comment on the PFA intervention they received. All interviews were conducted individually.

### 2.6. Analysis

Data from interviews were transcribed and coded. To meet our three objectives, the data was analyzed according to an inductive thematic analysis approach. This approach is particularly relevant when exploring a new field of knowledge. The codification process includes the following steps: preparation of raw data, careful and thorough reading, identification and description of categories, and refinement of categories (Braun and Clarke, 2006). As with other qualitative analyzes, the primary objective of this analysis was reduction, or synthesis, of the corpus. It was translated into significant units, or themes that are representative of the content gathered to address the research.

questions. This analysis allowed for the exploration of links and contrasts between the themes. The analysis proceeded as follows: two researchers developed a thematic tree based on the themes that emerged from the first five interviews transcribed and conducted with PFA respondents. The last author reviewed the suggested themes, and disagreements led to reexamining the data until the raters reached an agreement. After reaching a consensus on the themes, this thematic tree, which consists of organized and defined themes, was then applied to the remaining transcripts by the third author to all groups of participants. Subsequently, the fourth author performed double coding reaching a rate of 94% agreement. Our intercoder reliability was therefore considered sufficient to continue data interpretation. Themes regarding our research question were then analyzed to obtain a better and more complete understanding of our participants experiences of PFA. This process was done by all research members, assuring the credibility, the internal validity, of our analysis.

## 3. Results (semi-structured interviews)

Since our thematic tree, developed from interviews with PFA responders [R], did not require modification when applied to beneficiaries [B] and managers [M], themes will be presented jointly while distinctive features and verbatims for each group will be highlighted. Thematic analysis identified eleven themes (see Table 1).

**Table 1:** Emerging themes from semi-structured interviews.

1. PFA meets a long time need in the Quebec police workforce.
2. PFA offers easy access to psychological support and a rapid response to TEs.
3. Peers are in best position to enable openness in police officers and help them transition to second-level care.
4. Being a PFA provider is a natural continuation of supervisors’ role.
5. Responders’ proactivity is a crucial element to provide support.
6. Police officers are more open than before to talk about their emotions / difficulties.
7. PFA approach is useful, flexible and provides helpful tools.
8. PFA intervention meets beneficiaries’ needs and generates various positive impacts.
9. Lack of time and lack of responders constitute main challenges to the administration of PFA.
10. Potential reluctance to confide in a PFA responder in a position of authority or with whom the relationship is not optimal.
11. Being a PFA provider can be demanding and lead to concerns about this role.

### 3.1. PFA meets a long time need in the Quebec police workforce

For most participants, PFA responds to a deep-rooted need in the police workforce. Specifically, participants voiced that the nature of their work is emotionally demanding, that it is “easy to be thrown off balance,” and that they have personally witnessed the impact of such hardships (e.g., colleagues on medical leaves, suicides). Participants stated, “that there is really a problem that exists.” Also, some participants expressed that there has not been sufficient mobilization from the organization.

Since the beginning of this project, I say to myself “wow”, it was time for the SQ to get involved. It is a large organization which, I think, has seen over the years issues which are unfortunately related to staff well-being. And the best placed [to provide support] are their staff. I tell you, there are good people here… So yes, it was time for the organization to do something like this … I was the first to claim it when I got the invitation for this pilot, I said to myself: “finally, finally”. [R19]

### 3.2. PFA offers easy access to psychological support and a rapid response to TEs

Although certain procedures and resources are available in response to SQ police officers’ psychological distress, some participants expressed that resources are not tailored to their needs. For example, participants mentioned that access to emotional debriefings is not straightforward, and the delivery format is inadequate (e.g., group sessions offered by unknown civilians). Also, most beneficiaries stated that internal procedures do exists in response to TEs, but the focus is solely on the operational component of the event.

[PFA], it's the only approach, I could describe as human-oriented, focused on people. The only intervention, let's say psychological, social, that we patrollers have had up until now. Months after the event, we had several meetings, several debriefings, but always operational, strategic, nothing emotional. [B2]

In contrast to other resources, participants considered PFA to be highly valuable because it was offered by trusted and understanding peers. Thus, PFA responders were in a favorable position to notice difficulties and act quickly if needed. The implementation of PFA was perceived as an acknowledgment of members’ needs by the organization and a step toward a better tailored support.

PFA, it’s with people we know … we are there, we are close to them. I’m a supervisor so they have a lot more confidence in me than [operational] debriefing guys from all over the force. Us, we’re right here, in the field, we’re with them. I find that PFA [responders], we are much more out there than other resources … Traumatic events happen when I’m working, out there. Debriefings are done later, sometimes a week later… but when an event occurs, we need to deal with it, like, right now. [R20]

Even a mundane, reoccuring event can become…can lead to trauma. Sure there are events that are out of the ordinary that affect us, but sometimes they are not extraordinary enough for there to be a reference right away to the EAP or to request that a psychologist be dispatched for a debriefing. In these kinds of situations, if there is no PFA follow-up, then you are like left in a void. Then that doesn't mean it doesn't affect you, you know? So I think it's essential…PFA is becoming more and more essential at the in-between level. [R10]

### 3.3. Peers are in best position to enable openness in police officers and help them transition to second-level care

All three groups of participants described the police workplace as an environment where “ties are very strong” and where members are considered “family.” Participants mentioned that they rely on and trust each other. Due to shared experience, they also felt that their colleagues could understand them better than civilians. Responders stated that most civilians could not grasp nor cope with the horrific incidents faced by police officers. Therefore, the relationship of trust and mutual understanding among colleagues was perceived as beneficial for disclosure. Specifically, confiding in peers allowed honest and straightforward discussions without concerns about protecting others from disturbing content. Additionally, participants mentioned that the PFA responders working in the same unit as beneficiaries had great follow-up opportunities. According to responders, initial care provided by a person of trust would facilitate the transition to a “second level of care,” (i.e., to make a referral to a help resource, if necessary). In short, responders viewed themselves as “conveyor belts” in the helping process; a role they felt comfortable with.

Well of course between colleagues often there are things that we may both have experienced, so you are perhaps more comfortable going to talk about this to someone you know who experienced it. This feeling of mutual understanding, I find it’s better with a colleague than with someone from the outside who has no idea what you actually do for a living … Who doesn't know what it's like to untie a hanged person, who doesn't know what it's like to have a gun pointed at you or to find a dead child. I mean, if someone has just found a dead child, well, I know what it is, I know what he just went through. So reaching out is perhaps easier with a colleague than with an external counselor. [R26]

[PFA] I find that it's already a good way to de-dramatize the situations we are experiencing, then to perhaps, if we need help, to be more inclined to go further in the process. Me, as [a PFA responder], I'm like a first level, you know. It can lead people to confide more easily, to go to other levels. [R11]

### 3.4. Being a PFA provider is a natural continuation of supervisors’ role

The majority of PFA responders in this pilot project worked as supervisors for new recruits at Sûreté du Québec. Participants across all groups felt that it was strategically relevant for supervisors to act as PFA responders. When interviewed, supervisors reported being naturally inclined to advise and assist team members. Many also considered PFA training as an opportunity to acquire tools to efficiently support members of the organization. Further, supervisors encouraged the training of other members (e.g., patrol officers) as PFA responders, but only those with good interpersonal skills.

I think it's advantageous in the sense that we are supervisors, so it's our gang, it's our team, it's like our little family. How I see it, being a supervisor, you protect your family and you guide them so they can develop, become better. It’s relevant that it’s us who have this training since we know our people, we meet them on a daily basis, we see them go. So if there's something wrong, you often see it, especially if you have a good bond with your employee. So, you know, [being a PFA provider] it gives a nice way to find out more and then figure out what's wrong. [R26]

Basically, I would say that I do it in a somewhat natural way, it’s just that before, I didn't have the training. But you know, it goes without saying when you are a supervisor, you take care of your people and I was thinking maybe I'll be able to go get tools that will help in that. So, that’s what motivated me at first. [R21]

### 3.5. Responders’ proactivity is a crucial element to provide support

Most participants, across all groups, considered important for PFA responders to initiate contact with members when they see fit (e.g., during/after a disturbing event, when some form of distress is suspected). Different reactions can be observed when confronted with a PTE. A beneficiary describes the following:

After that [police] intervention, I was overwhelmed. But there is a bit of pride that kicks in. I want to continue, I'm here, I'm operational. So I liked the fact that it was him who approached me first (…) and just him to say: "No, go in your vehicle, take care of yourself first". Anyway, there wasn't much more I could do about the intervention at that point. So him, telling me "Look, it's not against you but just take the time to breathe by yourself, take it easy for a few minutes". Then there, it just made me realize that yes, there's no hurry, I can go take 2-3 breaths, take the time to come back to my senses. [B4]

When discussing long term follow up, participants described police officers as struggling to acknowledge the need for support and how to access it. Thus, many participants perceived responders’ proactivity as an important strength of the PFA program compared to other resources (e.g., employee aid program).

The responder, from my point of view, I see more that he will take the initiative, that he will go to see the person: if he is not well, he will notice it. While the EAP, external resources, if you don't call them, they won't call you. (…) So the responder, being a peer, he is closer. I find that the advantage with PFA is that it is us who will take the lead. That's one more strength there. [R9]

[PFA responders should be] proactive, I think, because we’re all stubborn people who think we’re strong and indestructible, so yeah, that’s the way it is with us. We end up after a couple of years with members who fall in battle, then we realize that it’s events that happened a few months ago, even a few years ago, that affect us. So, no choice to be proactive because we’re fools, we don’t go looking for help until it’s too late (laughs). [G1]

### 3.6. Police officers are more open than before to talk about their emotions/difficulties

Many participants provided a similar description of policing culture. Specifically, it was described as praising a though and strong demeanor among members. These perceptions may represent barriers to pursuing psychological help. However, most participants reported that the police mentality has considerably evolved in recent years. Namely, the new generation of police officers were described as open, aware, and comfortable to discuss mental health issues. As one responder described: “basically, all police officers experience the same anxieties, the same fears,” but that the “old police” is typically more reluctant to talk about it for fear of appearing weak and being judged. However, even the older generation tends to open because, even if they may not openly acknowledge it, they know police work can be emotionally demanding, even damaging.

It's true that the police mentality has changed a lot in the last few years, and since I joined the police. You know, it's much more open. People talk more about their emotions. I still work with “old police” colleagues that were hired because of their big hands, because they were six foot four. Back then, we didn't talk about those things, I would tell you. In those days, forget it, [a peer support program] would never have worked. Offer a colleague to go and chat about their emotions? That would have been, like, asking for trouble! For sure, today, there is much, much more openness from everyone to talk about these matters. The proof is that everyone is affected when a tragedy occurs. We’ll react by talking about it. Times have changed, you know, we’re at a different place now. [R2]

According to responders, members’ interest in the PFA program demonstrated a positive shift in mentality within the organization. Generally, participants felt that police officers were grateful that an assistance program was made available for them. Although some participants clearly voiced their appreciation, others provided brief responses or jokes. Responders reported that these latter reactions should not be interpreted as indifference. Rather, some participants may be hesitant to comment on mental health issues, especially in group situations.

How did my team respond to the program? Well, I think a bit like typical police officers. Like: “Oh. OK". (laughs) … Like, even though the mentality tends to change, the fact remains that we are police officers, that everything is fine all the time and there is nothing that affects us. […] But when you do it “one on one”, they are more open. In a group, I found the reaction kind of aloof, but in the sense that they are not going to have a party about it. Like “It's good that we're implementing this, it's good that this training is available”. It was positive, but I wouldn’t say they were very expressive. [R26]

I explained to my team what the PFA program consisted of and what we had acquired in the training. There was a big openness about this. But there is absolutely no doubt in my mind that this has to do with this particular station having a lot of young police officers. So, being younger, the reality of mental health, post-traumatic stress, you know, there is more acknowledgement. So yeah, it was very well received. [R7]

### 3.7. PFA approach is useful, flexible and provides helpful tools

Responders in this study expressed high satisfaction regarding PFA for various reasons. First, many considered the training useful. Second, they believed the training increased confidence in their ability to support fellow colleagues. Responders reported being better equipped to detect, understand, and act upon psychological distress. Third, multiple references were made regarding the benefits of the adapted PFA intervention. For example, responders appreciated being able to intervene quickly in a variety of situations. The PFA approach was also characterized as easy to use, namely because it provided responders with clear action points. Further, PFA was viewed as more relevant and respectful of individual pace than other trainings. Finally, responders valued having access to useful materials (e.g., reminder/checklist, intervention report canvas) to plan upcoming interventions or to review completed sessions.

I liked [the PFA training], I thought there were many useful tips such as taking the police officer to a patrol car to make him feel better, to take him away from the scene of an accident. And those kind of tips, you can apply them to almost everyone. Like, again, when there is an accident, sometimes you need to ground the victims, calm them down so they can talk to you. So, yeah, these are great tools that I could even use with people other than my colleagues. The PFA approach, I feel it's tailored to our reality. And as supervisors, I think it fits with the way we work. [R5]

There is a very good level of satisfaction because there are different forms of support that are available [to the PFA providers]: co-development sessions, documentation that we can read or give out. We have material to work with. That’s a point that is extremely positive…And what we learn about how to be good listeners: try not to judge the person, try to understand…and empathy, not sympathy, because if you cry with the person, well, you're not really helping. And also, how to structure and prepare your meeting, what tools you are going to offer the person, etc. You know, have a basic idea of what you plan to do, well, like an intervention plan. [R1]

### 3.8. PFA intervention meets beneficiaries’ needs and generates various positive impacts

Beneficiaries who were interviewed in this study expressed satisfaction regarding the PFA approach, particularly because it focused on their needs. Therefore, beneficiaries viewed interventions as genuine demonstrations of concern for their well-being. These interactions allowed them to positively reflect on their situation. Specifically, the PFA intervention addressed both conscious and unconscious needs of trauma exposed officers. A few examples included the following: attention to basic needs (e.g., taking the time to drink/eat), taking time to decompress/regain one’s senses, ventilate (i.e., speak freely), take time to assimilate disturbing events and finally, have access to things useful for their well-being (e.g., documentation, help resources).

The PFA approach had several positive effects for beneficiaries. First, they reported feeling validated by trusted peers who have shared experience. In turn, the intervention helped beneficiaries normalize their emotions and reactions. Finally, it helped to put the situation into perspective.

When the first person you talk to is your colleague, it allows you to normalize your emotions, you say to yourself: “well, maybe it is normal that I’m shaken like that.” [B3]

The PFA concepts… just taking the time to be reassuring, to say that it’s normal. Just this: “Look, these reactions may happen to you in the next few days, you may have bad dreams … That doesn’t mean you’re in post-trauma. We’re not there yet, but these are normal reactions.” Just that, sometimes, I find it relevant to mention it when it’s just happened because, you know, me, it’s happened to me, to have something that has shaken me, but nobody has said to me, "Hey, you know, it’s normal." I figured it was normal, that I’m probably not the only one, but getting that kind of heads up, I think, that can be good. [R8]

Several beneficiaries reported that responders provided information (i.e., through discussion or documentation) about reactions that can occur in the context of emotionally demanding work. This allowed beneficiaries to remain attentive to their reactions and their needs.

[The responder] gave me documentation that I still have at home. A sheet with phone numbers and another with reactions that I had to monitor, things that could happen, like hypersensitivity, lack of sleep … And then to say, look at these reactions, it's normal. Except that if you keep having these kinds of reaction, you should go get a little more help than…You should talk to someone. And basically, I was satisfied with what he offered me. Me, I made a call to the Vigil. [B4]

In addition to the benefits provided by the PFA intervention, the implementation of such a program within the organization was reported as reassuring for its members. Participants commented that the program created a sense of hope.

I felt he listened, and he knew what I was talking about. He understood me, he was ready to help me, that's what gives me a feeling of hope, to say to myself “look even if I'm going through difficult situations, there is help” (…) We deal with situations, then once in a while, we hear about a colleague who has committed suicide. That’s like, hard to swallow because I live the same situations as him, it's scary at times. To just say look, there are things that are put in place, trainings, people who are there to help, that’s also reassuring. Even if I go through difficult situations, there is a way out. [B4]

### 3.9. Lack of time and lack of responders constitute main challenges to the administration of PFA

For several participants, mainly responders and managers, the lack of time was identified as a barrier to the administration of PFA. A few responders mentioned being overworked, and unable to consistently add PFA to their workload. Typically, there was a single responder per team or even per police station. Hence, if a responder was not working during a major event, PFA could not be delivered in an adequate time frame. Moreover, PFA administration is not recommended if both the responder and their team are involved in the major event.

Because often the more major an event, the more police officers need psychological care and the more the supervisor (PFA responder) has tasks and involvement. Basically, everything increases exponentially with the severity of the event. And basically, the bigger [the event], the more police there are, the less time I have. So there's like a barrier there which is relatively major to say I have to give my members time there, it's urgent, but at the same time, I have a lot of tasks to do and I have to do them too. So that brings a limit, pretty quickly. [R25]

Additionally, responders stated that the involvement of the Internal Investigations Bureau distracts them from administering PFA. Participants across all groups said they are in favor of having a greater number of responders trained within their units.

### 3.10. Potential reluctance to confide In a PFA responder in a position of authority or with whom the relationship is not optimal

Some responders raised the concern that police officers may be more reluctant to discuss psychological distress with colleagues in a position of authority. This reluctance is explained by desire to maintain a positive reputation (e.g., strong, competent) and by fear that disclosure will cause negative consequences (e.g., affect performance evaluation). However, reluctance would emerge from lack of trust and tense relationships with superiors. Also, a perceived lack of interpersonal skills in responders would negatively affect decision to disclose. Some participants also mentioned that team members do not necessarily have equal affinity with their supervisor. In addition, even if the supervisor is appreciated, certain one-off events can temporarily hinder the relationship. Consequently, almost all participants in this study suggested that members should have the ability to choose between at least two PFA responders.

I think it should also include patrollers, those who are comfortable talking to others. They can also help. […] Sometimes it's harder to confide in a boss. It depends on everyone, but you know, equal to equal, sometimes it can help. In front of your boss, admiting that you have problems, it’s like … you always want to look good, so that might be a small obstacle to the program. [R9]

It can depend a lot on the relationship you have with the [supervisor/PFA responder] and that can change from time to time. For example, I am the manager of my unit. I get along with everyone, but sometimes it can be a little less friendly because I need to call them to order, things like that. So, let's say on the same day, there's a big event and there is a need to administer PFA. I'm not sure the timing would be perfect. So it might be necessary to have more than one PFA provider per unit. [R3]

### 3.11. Being a PFA provider can be demanding and lead to concerns about this role

Offering psychological support can sometimes be an ambiguous process and some of the participants commented on this challenge. For example, a responder wondered if he has done enough to ensure the safety of the beneficiary and how this can be determined. Other responders found that offering follow-ups for up to a month is strenuous. However, some responders had blurred lines between peer support and professional help. Other responders felt uncomfortable discussing help resources with beneficiaries due to a lack of necessary knowledge.

For everything that is on the spot, I think [PFA] is wonderful. But for the more long-term part…Maybe we need to tell people that we're not professionals, that we don't do therapy. We are peer helpers. We focus on what can be done now, at the time of the traumatic event, a few hours later, one week after the event. But after that, who do I pass the puck to? To say: “Look, you're doing better, but I still feel you're fragile, what do you think about keeping this up, and then someone can come in to support us”. But a month later, I find that this is beyond our role as PFA responders. [R19]

A few managers expressed concerns about the additional pressure as well as the emotional burden placed on PFA responders.

The challenge we have at the Sûreté is always to have people out there on the field… We’re short of staff. like everywhere else. This is our stake. So everything goes quickly, and then we ask more and more of our police officers (…). So the issue is there for the supervisor as well. I find that it is also a lot for one person, to tell him: "You are the lookout, that’s your role." You know, it can be pressuring for some members, to be the bearer like that. [M6]

Depending on the problem known or experienced by the member in need, it remains that the responder is a police officer, not a professional trained with a PhD in psychology. So, yes, there are limits to that, and then there are limits as to not exhaust the responder. So he doesn’t transpose that and bring it home either. [M3]

## 4. Discussion

This study aimed to evaluate the feasibility of PFA as an early intervention following an exposure to a TE among police officers in Quebec, Canada. In this study, PFA was provided by supervisors. An inductive thematic analysis was used to assess the demand, practicality, and acceptability of PFA. First, the findings revealed that PFA met the needs of the participants. Second, analysis showed that PFA was successfully adapted to policing reality and culture. Third, implementation occurred without major issues. Finally, the program had positive impacts on individuals and on their perception of organizational support. Specifically, PFA destigmatized mental health issues and renewed a sense of hope among police personnel.

### 4.1. Demand

Assessing demand can be done by examining the extent to which a new program or intervention is likely to be used (Bowen et al., 2009). One factor contributing to the utilization of a program is its capacity to meet the needs of its beneficiaries. Our findings revealed that PFA met longtime needs within the organization, as participants expressed that this intervention satisfied an urgent need among Quebec’s police forces. While the consequences of organizational stress related to policing culture have been intensively studied in recent years, it appears that interventions aiming to mitigate PTSI symptoms are still inadequate to meet the needs of workers. Indeed, research has shown that being exposed to traumatic events on an ongoing basis can have a significant impact on mental health (Purba and Demou, 2019), and that police officers are particularly vulnerable to PTSIs (Carleton et al., 2019; Purba and Demou, 2019). The introduction of terminologies like “complex police spiral trauma” (PCST; Papazoglou, 2013) and “cumulative career traumatic stress” (CCTS; Marshall, 2006) expresses the emotional difficulties of police work (Violanti et al., 2017; Padilla, 2020). As such, several interventions have been tried to reduce PTSI symptoms among first responders in the past (Carleton et al., 2020). Therefore, participants of the present study expressed that PFA is the first intervention to adequately address the emotional impact of TEs experienced during their work. Consequently, the implementation of PFA was perceived as a step toward better-tailored support as well as an acknowledgement of their needs on the part of the organization. This is especially important because research has shown that officers usually do not ask for help until they are experiencing substantial stress (Daniel and Treece, 2022), but make greater use of available resources when they feel that their organization supports them (Tucker, 2015). Although the number of PFA interventions was not tracked during the study, all participants stated that this intervention addressed an unmet need within the organization that had existed for several years. This suggests that demand may increase if the organization decides to implement the program throughout their organization.

### 4.2. Practicality

Assessing practicality involves examining the extent to which a new program or intervention can be carried out with participants using existing means, resources, and circumstances (Bowen et al., 2009). Findings revealed facilitators and constraints that may affect the practicality of PFA among police officers. First, respondents stated that PFA was very easy to use. In fact, most of them praised the training’s eight-step reference guide for interventions. They also stated that PFA felt more natural to them than other forms of intervention because it was like what they would normally do. Thus, the close relationship maintained between the research team and the organization during the adaptation of both the training and the intervention appears to have been crucial for the PFA’s effective operation and the participants’ adherence. Another crucial element enabling police officers to receive PFA was the responder’s proactivity. This observation adds an important point to the recommendations provided by Forbes et al. (2011) about the deployment of PFA within high-risk organizations. Indeed, some organizational cultures, like those of police forces, carry numerous stigmas regarding mental health issues (Soomro and Yanos, 2019; Wheeler et al., 2021). Even though participants mentioned that the SQ police mentality has changed in recent years, the “strongman” culture still constitutes an obstacle to seeking psychological help within this organization. Similarly to Hofer and Savell’s (2021) findings, many participants interviewed in the present study felt that police officers are reluctant to seek mental health care because of their pride and for fear of being perceived as weak. Consequently, most participants within all three groups considered that PFA responders should take the lead in offering assistance and considered that PFA’s proactivity (through its first action of contact and engagement) is a strength when compared to other resources.

Findings also indicated that responders and beneficiaries were generally comfortable with each other’s roles. Responders felt that being a PFA provider was a natural continuation of their supervisor’s role, as it is part of their mandate to assure the physical and psychological well-being of their colleagues. As for the beneficiaries, they mentioned being comfortable seeking help from their responders, even if they were their immediate supervisors, but placed emphasis on the fact that whoever acts as a PFA responder must possess good interpersonal skills and be trustworthy. This finding is consistent with Heffren and Hausdorf (2016) study, reporting that in the context of peer support, police officers were sharing personal information with their direct supervisors only when there was a strong, reciprocal relationship between them.

Despite those findings, some beneficiaries suggested that more responders, from various hierarchical positions, should be trained to ensure the sustainability of the intervention within the organization. For instance, this study revealed that supervisors generally have a heavy workload. Moreover, some responders mentioned perceiving their role as time consuming, ambiguous, and emotionally demanding. These obstacles are in line with the research on the possible consequences associated with helping others (McCann and Pearlman, 1990; Figley, 1995; Stamm, 2012), as well as with the current understanding of police work as emotionally challenging in nature (Carleton et al., 2019). An appropriate response to these obstacles could contribute to the establishment of a trauma-informed care approach within the organization, improve the well-being of more officers, including the responders, and promote the organization’s self-efficacy (Chandra et al., 2014; Wang et al., 2021). To do so, organizations should train additional responders, promote the expression of responders’ concerns, and offer them adequate support such as individual coaching, group supervisions, and refresher training on PFA and mental health issues (Brillon, 2013). These recommendations are congruent with the Dynamic Sustainability Framework (DSF), which was developed for the implementation of health interventions in organizations (Chambers et al., 2013), as well as the current literature on PFA’s implementation in organizations. Research effectively showed that PFA training gives responders the confidence to use the intervention with prospective beneficiaries even if they were not trained in mental health (McCabe et al., 2011), and that the adherence of Emergency Medical Services workers as PFA-responders was based on their confidence and ability to provide an adapted intervention as well as their feeling of being adequately equipped (Tessier et al., 2021).

### 4.3. Acceptability

Assessing acceptability can be done by examining the extent to which a new program or intervention is judged suitable, satisfying, and appropriate to responders and beneficiaries (Bowen et al., 2009), when various elements, such as the program’s conformity to the organizational culture and the perceived impacts on the organization are taken into consideration.

Our findings revealed that the PFA-SQ approach and program were considered well-tailored and acceptable to the organization’s environment and culture, namely because it met various needs offered by proximity support. In this case, peer support provided by in-house PFA responders was praised because it constituted quick and immediate support by individuals that are deemed credible, trustworthy and to whom officers could relate to. Participants strongly believed that colleagues are in the best position to facilitate openness among police officers, especially compared to outside resources. Because of the proximity that is characteristic of police officers working within a dedicated unit, the PFA responders reported they were in a privileged position to detect difficulties and to offer follow-ups as needed. Also, participants expressed that with peers, compared to family members, they do not need to worry that their conversations will have a disturbing effect on them. This finding is different from what Carleton et al. (2019) found when they looked at what 4,020 Canadian public Safety workers thought about their access to professional and non-professional help. In this study, even though most participants reported access to professional and non-professional support, 74% stated that the first person they would turn to would be their spouse. Carleton et al. (2019) pointed out that these results remained consistent with reports stating that public safety personnel perceived substantial stigma and were unwilling to get support from colleagues but were willing to reach out to family and friends (Oliphant, 2016; Ricciardelli et al., 2018a,b). The following reasons might be considered to explain discrepancies between these results. First, our sample may not reflect the opinion of the majority of SQ’s police officers due to the sampling method we used. Second, participants included in Carleton et al. (2019) sample was not limited to police officers. Third, cultural differences between study participants may have had a role in the observed disparities, as their sample was composed of English-Canadians and ours of French-Canadians. Lastly, Carleton et al. (2019) did not specifically assess the support participants felt they could have in response to work related traumatic events, but rather looked at the support they felt they could have if they “needed help managing their mental health.” This difference could have generated different results.

### 4.4. Implications

From a practical standpoint, the administration of PFA through peer support can contribute to organizations’ self-sufficiency in mental health assistance, as police officers are generally uncomfortable seeking outsiders’ help. This study also provided key information to further refine the PFA program within the police culture and ensure its sustainability. Thereby, this study has allowed for the development of recommendations to facilitate future deployments. First, it would be essential to train more responders so that beneficiaries have a selection of responders to choose from (e.g., colleagues or superiors). Second, PFA responders should be provided with adequate support such as coaching, group supervisions and refresher training. Third, sufficient visibility should be given to the PFA program using various dissemination methods but prioritizing person to person contact. Efforts in clarifying particularities and differences between various resources available should also be made to help workers find what best fits their needs.

### 4.5. Limitation

To properly interpret the findings of this study, limitations must be considered. Although data saturation appears to have been reached, it is possible that more themes would have emerged if the sample size had been larger, especially from the beneficiaries group. Second, the convenience sampling and the fact that the intervention received minimal unfavorable remarks from participants may reflect a selection bias. Lastly, qualitative studies do not seek generalization. The findings of this study should therefore only be regarded within the context of a peer support structure among police officers.

### 4.6. Conclusion

In conclusion, the implementation of a PFA’s peer intervention within the culture of policing is an approach that should be considered for mitigating the negative effects associated with exposure to traumatic events. PFA offered by colleagues echoes the call for stepped care and personalized health models intervention (Lord et al., 2022). Also, peer support can be utilized across settings and is designed to adjust to individual needs (Shea et al., 2022). The peer support model examined in this study was built using a co-construction model tapping into expertise from both researchers and key members of the organization. We are confident this has contributed to its feasibility, namely its high acceptability. Future studies should favor this collaborative framework to ensure PFA’s sustainability within the Sûreté du Québec and possibly within other police organizations.

## Data availability statement

The datasets presented in this article are not readily available because French verbatims of interviews accessible on demand. Requests to access the datasets should be directed to SG, s.geoffrion@umontreal.ca.

## Ethics statement

The studies involving human participants were reviewed and approved by CIUSSS de l’Est-de-l’Île-de-Montréal ethics committee (#2022-2673). The patients/participants provided their written informed consent to participate in this study.

## Author contributions

SG conceptualized the study, obtained funding, conducted the PFA training, supervised the data collection, analyzed the data, and wrote the article. M-PL conducted the data collection. M-PL, EB, FB, and VA analyzed the data, and wrote the article. CG conceptualized the study, obtained funding, and wrote the article. All authors contributed to the article and approved the submitted version.

## Funding

This study was funded by a SSHRC 892-2020-3010 grant and a career award to the first author from the FRQ-IRSST #268274.

## Conflict of interest

The authors declare that the research was conducted in the absence of any commercial or financial relationships that could be construed as a potential conflict of interest.

## Publisher’s note

All claims expressed in this article are solely those of the authors and do not necessarily represent those of their affiliated organizations, or those of the publisher, the editors and the reviewers. Any product that may be evaluated in this article, or claim that may be made by its manufacturer, is not guaranteed or endorsed by the publisher.

## Footnotes

^1^ https://www.publicsafety.gc.ca/cnt/mrg3nc-mngmnt/mrgnc-prprdnss/ptsi-en.aspx

^2^ https://www.publicsafety.gc.ca/cnt/rsrcs/pblctns/2019-ctn-pln-ptsi/index-en.aspx

^3^ https://www.who.int/publications/i/item/9789241548205

^4^ https://www.sq.gouv.qc.ca/organisation/mission-vision-valeurs/
