# Supplementary material for: A feasibility study of psychological first aid as a supportive intervention among police officers exposed to traumatic events
Source: Front Psychol. 2023 Mar 13;14:1149597. doi: 10.3389/fpsyg.2023.1149597 (PMC10040866; doi:10.3389/fpsyg.2023.1149597)
Supplement: Supplementary file 1 [file Data_Sheet_1.docx]

**APPENDIX 1. INTERVIEW GRID FOR PFA RESPONDERS**

- What motivated you to become a PFA responder?
- After receiving the PFA training, how was your first experience as a PFA responder?
- What do you think about psychological first aid ?
  - …as a way to offer support (in general)?
  - …as a way of providing support in your workplace (policing environment / police officers)?
- In your opinion, is PFA adapted to your work environment?
  - How so?
  - Are there any potential changes that could make it more suitable for your work environment?
- What do you think about support being provided by co-workers?
  - Are there benefits?
  - Disadvantages?
- Do you think there is a need for PFA within your organization?
- Now, can you tell me more about the PFA interventions you carried out?
  - How did it go? / How did your colleague(s) react?
  - How did you experience it? / How did you feel? (any challenges or obstacles?)
  - What impact did PFA have on your colleagues?
- What is your level of satisfaction with the PFA approach?
- Do you think there are any obstacles or challenges to the implementation of PFA within the Sûreté du Québec? (What are those obstacles / challenges?)
- What could facilitate or improve the implementation of PFA into your workplace?
- How could we make the PFA program better?

**APPENDIX 2. INTERVIEW GRID FOR PFA BENEFICIARIES**

- Can you tell me about the context in which you received psychological first aid from your colleague?
  - Would you be able to tell me what were your needs at that time?
- Did your colleague offer you advice or recommendations? …Documentation?
  - Which ones have helped you the most? Why?
- What do you think about psychological first aid ?
  - …as a way to offer support (in general)?
  - …as a way of providing support in your workplace (policing environment / police officers)?
- In your opinion, is PFA adapted to your work environment?
  - Do you feel the support you received was suited to you and/or to your needs as a police officer? How so?
- What do you think about support being provided by co-workers? (Any reluctance or concerns?)
  - Are there benefits? Disadvantages?
- Did PFA (the support you received) meet your needs? In what way(s)?
- What is your level of satisfaction with the PFA approach?
- How could the intervention/type of support you received be improved?
  - Are there things you wish had been done differently?
  - ...things that would have better suited your needs?
- Do you think there is a need for PFA within your organization?
- Do you think there are any obstacles or challenges to the implementation of PFA within the Sûreté du Québec? (What are those obstacles / challenges?)
- What could facilitate or improve the implementation of PFA into your workplace?
- How could we make the PFA program better?

**APPENDIX 3. INTERVIEW GRID FOR MANAGERS**

- What do you think about psychological first aid ?
  - …as a way to offer support (in general)?
  - …as a way of providing support in your workplace (policing environment / police officers)?
- In your opinion, is PFA adapted to your work environment?
  - How so?
  - Are there any potential changes that could make PFA more suitable for your work environment?
- As a manager, can you tell me about your experience with the implementation of PFA within your unit?
  - Any challenges, concerns?
- What do you think about support being provided by co-workers?
  - Do you see any benefits? Disadvantages?
- Do you think there is a need for PFA within your organization?
- What is your level of satisfaction with the PFA approach?
- Do you think there are any obstacles or challenges to the implementation of PFA within the Sûreté du Québec? (What are those obstacles / challenges?)
- What could facilitate or improve the implementation of PFA within the Sûreté du Québec?
- How could we make the PFA program better?
